# EETs reduces LPS-induced hyperpermeability by targeting GRP78 mediated Src activation and subsequent Rho/ROCK signaling pathway

**DOI:** 10.18632/oncotarget.17331

**Published:** 2017-04-21

**Authors:** Ruolan Dong, Danli Hu, Yan Yang, Zhihui Chen, Menglu Fu, Dao Wen Wang, Xizhen Xu, Ling Tu

**Affiliations:** ^1^ Department of Geriatric Medicine, Tongji Hospital, Tongji Medical College, Huazhong University of Science and Technology, Wuhan, Hubei, 430030, China; ^2^ Institute of Integrated Traditional Chinese and Western Medicine, Tongji Hospital, Tongji Medical College, Huazhong University of Science and Technology, Wuhan, Hubei, 430030, China; ^3^ The Institute of Hypertension and Department of Internal Medicine, Tongji Hospital, Tongji Medical College, Huazhong University of Science and Technology, Wuhan, Hubei, 430030, China

**Keywords:** CYP450, 2J2, vascular permeability, Rho-ROCK, LPS

## Abstract

Integrity of endothelial barrier is a determinant of the prognosis in the acute lung injury caused by sepsis. The epoxyeicosatrienoic acids (EETs), metabolites of arachidonic acid, exhibit protective effects in various pathogenic states, however, whether EETs play a role in endothelial barrier enhancement and the involved mechanisms remain to be investigated. Here, we show that increased EETs level by endothelial specific cytochrome P450 epoxygenase 2J2 over-expression and soluble epoxide hydrolase (sEH) inhibitor TPPU reduced lipopolysaccharide-induced endothelial hyper-permeability *in vivo*, accompanied by improved survival of septic mice. In addition, sEH inhibitor AUDA and 11,12-EET also decreased endothelial hyper-permeability in the *in-vitro* study. Importantly, the relative mechanisms were associated with reduced GRP78-Src interaction and ROS production, and subsequently reduced RhoA/ROCK activation, and eventually decreased VE-cadherin and myosin light chain (MLC) phosphorylation. Thus CYP2J2-EETs is crucial for RhoA-dependent regulation of cytoskeletal architecture leading to reversible changes in vascular permeability, which may contribute to the development of new therapeutic approaches for pulmonary edema and other diseases caused by abnormal vascular permeability.

## INTRODUCTION

The inner surface of microvessels is lining by endothelial cells, which form a semipermeable barrier that actively participates in blood–tissue exchange [1]. Compromised vascular barrier function is involved in many pathological conditions including tumor metastasis and acute lung injury as well as diabetes [2, 3]. Increased lung vascular permeability and severe lung inflammation are two key features of acute lung injury (ALI) in septic patients, which typically develop in concert, leading to progressive deterioration of lung function [4]. The integrity of the endothelial cell (EC) monolayer directly determines lung vascular permeability since endothelial barrier between the vessel lumen and underlying alveoli mediated the transmigration of blood cells and macromolecules and maintain fluid homeostasis of lung [5]. In order to develop effective therapeutic strategies, we need to further explore the molecular mechanisms underlying pathogenic conditions related to microvascular hyperpermeability.

There are mainly two different routes by which the plasma proteins and solutes are transported across the endothelium: one transcellular, which involves the formation of transport vesicles, and the other paracellular, which requires disruption of the adherens junctions between 2 adjacent endothelial cells [6]. Solutes with radius of no lager than 3 nm can pass across the endothelium through paracellular routes [6, 7], while large molecular such as albumin can only be selectedly transported in form of vesicle by transcellular way [6, 8]. Although transcytosis contribute to basal permeability of the endothelium, paracellular flux of plasma fluid and proteins has drawn great attention because of its critical role in vascular inflammation associated diseases and injury. Cell junctions are mainly composed of tight junction, adherens junction and gap junction. Among the three types of junctions in the vascular endothelium, the tight junction and adherens junction are the best characterized functioning in mediating cell–cell adhesion [1]. Except that in some barrier-restricted organ such as blood–brain barrier where tight junction dominants, most vascular junctions is composed of adherens junctions. Thus initiating mechanistic events that stable adherens junctions is crucial to enhance barrier stability,

Vascular endothelial cadherin (VE-cadherin, also known as cadherin 5), the major type of inter-endothelial junctions, is a trans-membrane receptor whose extracellular domain homophilically binds to the extracellular domain of another VE-cadherin from an adjacent cell and whose intracellular domain is anchored to the cell cytoskeleton via a family of actin-binding proteins called catenins (α, β, γ and p120 catenins) [9]. Phosphorylation and internalization of VE-cadherin, and reorganization of the actin cytoskeleton into stress fibers, thus applying mechanical forces to AJs that break apart the junctions are responsible for increases in vascular permeability [6]. Any agent capable of inhibiting VE cadherin and myosin light chain (MLC) phosphorylation may effectively decrease vascular permeability that induced by inflammatory agents [10].

Cytochrome P450 epoxygenase 2J2 (CYP2J2), which is abundantly expressed in the vascular system especially in endothelial cells, transforms unsaturated fatty acid such as arachidonic and linoleic acids to various biologically active compounds, including epoxyeicosatrienoic acids (EETs) [11, 12]. EETs are further metabolized by soluble epoxide hydrolase (sEH) to DHET that are much less biologically active [13]. The CYP epoxygenases and sEH are expressed and metabolically active in various tissues and cell types, including endothelial cells and cardiomyocytes [14, 15], however, the physiological potency of so abundant CYP expression on vascular bed remains largely unknown. We used to take EETs as endothelial derived hyperpolarization factor and observations reveal that EETs exert anti-hypertension protection [16]. Recently, studies reported that CYP2J2 and its metabolites EETs protected against lung ischemia/reperfusion injury via the effects of anti-inflammation and anti-apoptosis of vascular endothelial cells [17]. Evidence in the literature suggests that sEH inhibitor AUDA reduced LPS-induced mortality via suppressing acute inflammation [12]. However, whether CYP2J2 and its metabolites EETs protects against LPS-induced acute lung injury and the relative mechanisms remains to be investigated.

There are another study showing that female ECs are less susceptible to ischemic brain injury is partly due to the lower level of sEH expression and higher EETs, which suggested that inherent differences in EC susceptibility to ischemia may in part underlie the differences in acute vascular responses [18]. Since the integrity of ECs is an important determinant of brain tissue perfusion after cerebral ischemia [18], we therefore hypothesized that CYP2J2 overexpression would attenuate microvascular permeability in a murine model of LPS-induced acute lung injury. In this study, the effects and potential mechanisms of EETs on microvascular hyperpermeability induced by LPS challenge were investigated by using *in-vivo* and *in-vitro* studies

## RESULTS

### EETs reduced LPS-induced mortality

**Figure 1:**
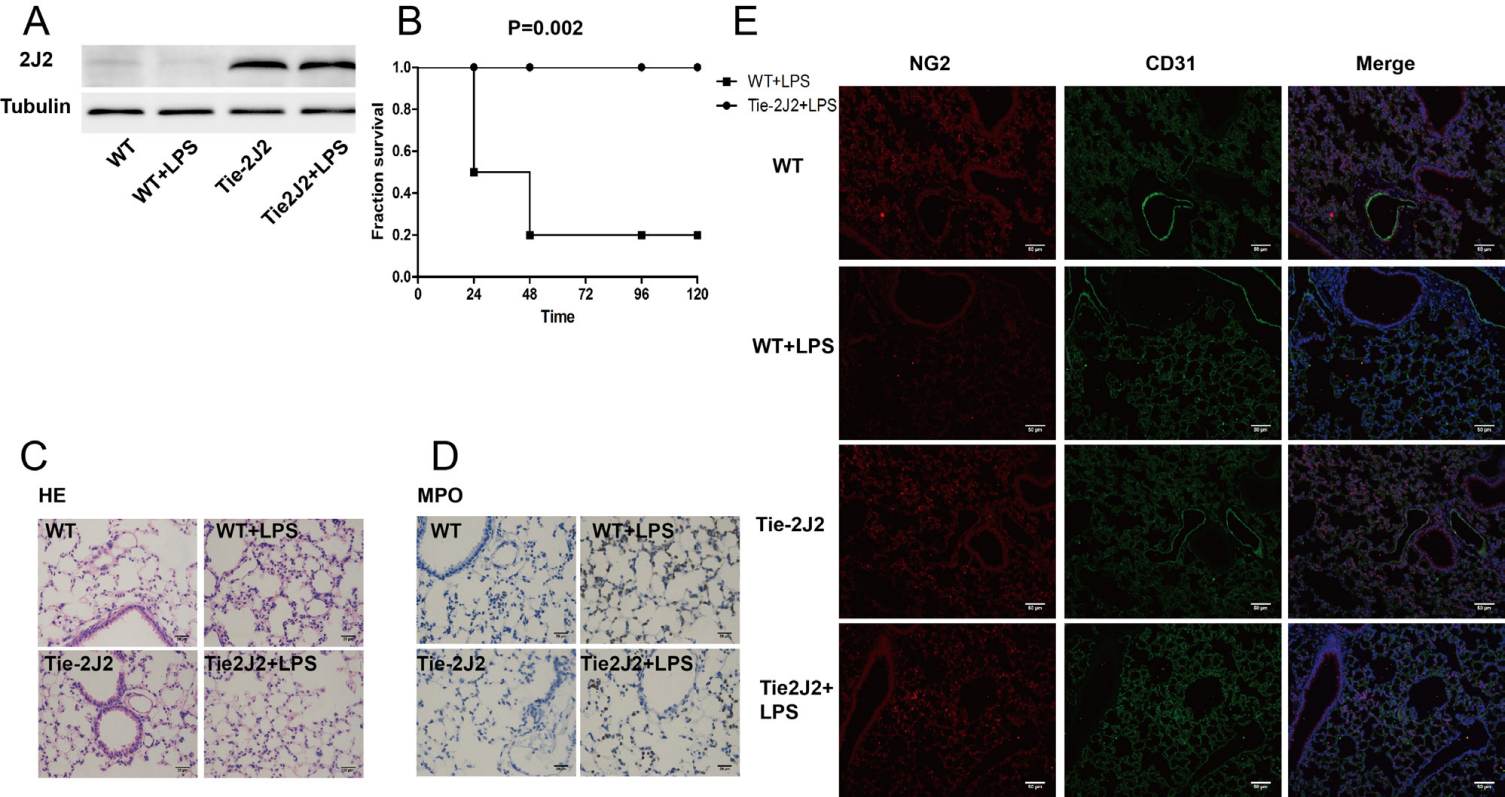
CYP2J2 overexpression reduced LPS-induced mortality (**A**) recombinant human 2J2 expression in WT and transgenic mice; (**B**) survival curve of WT and 2J2 transgenic mice in 96 hours after LPS treatment; (**C**) HE staining of lungs that indicates leukocytes infiltration; (**D**) immunohistochemistry staining of lung that indicates neutrophils infiltration; (**E**) immunofluorescence staining of pericytes that marked NG2. In survival curve test, 10 mice were used in each group, while for the other test, *n* = 5.

In order to evaluate the protective effect of CYP2J2 against LPS induced sepsis, endothelial specific 2J2 transgenic mice were used (Figure 1A). LPS challenge (15 mg/kg) resulted in 80% mortality in WT mice, while all CYP2J2 transgenic mice survived as shown in Figure 1B. Histological examination of lung tissue from LPS-treated mice revealed increased infiltration of white blood cells into the lung interstitium determined by HE staining and MPO immunohistochemistry, which was attenuated by CYP2J2 overexpression as shown in Figure 1C and 1D. Vascular endothelial cells are supported by mature pericytes. Loosen attach of immature pericyes with endothelial cells or loss of pericytes cause destabilization of vessel structure and impairement of endothelial barrier function [19]. We observed that pericytes that was marked by NG2 were greatly reduced by LPS treatment, and this effect was partly reversed by CYP2J2 overexpression (Figure 1E).

In addition, in order to increase the endogenous EETs level, sEH inhibitor TPPU was used. As expected, TPPU treatment markedly increased survival of septic mice (Supplementary Figure 1A) by suppressing leukocytes infiltration into the lung tissues characterized by decreased MPO expression and activity in lung tissues as shown in Supplementary Figure 1B, 1C and 1D. These data demonstrated that EETs significantly inhibited the progression of sepsis induced by LPS treatment.

### EETs decreased lung hyperpermeability induced by LPS challenge

**Figure 2:**
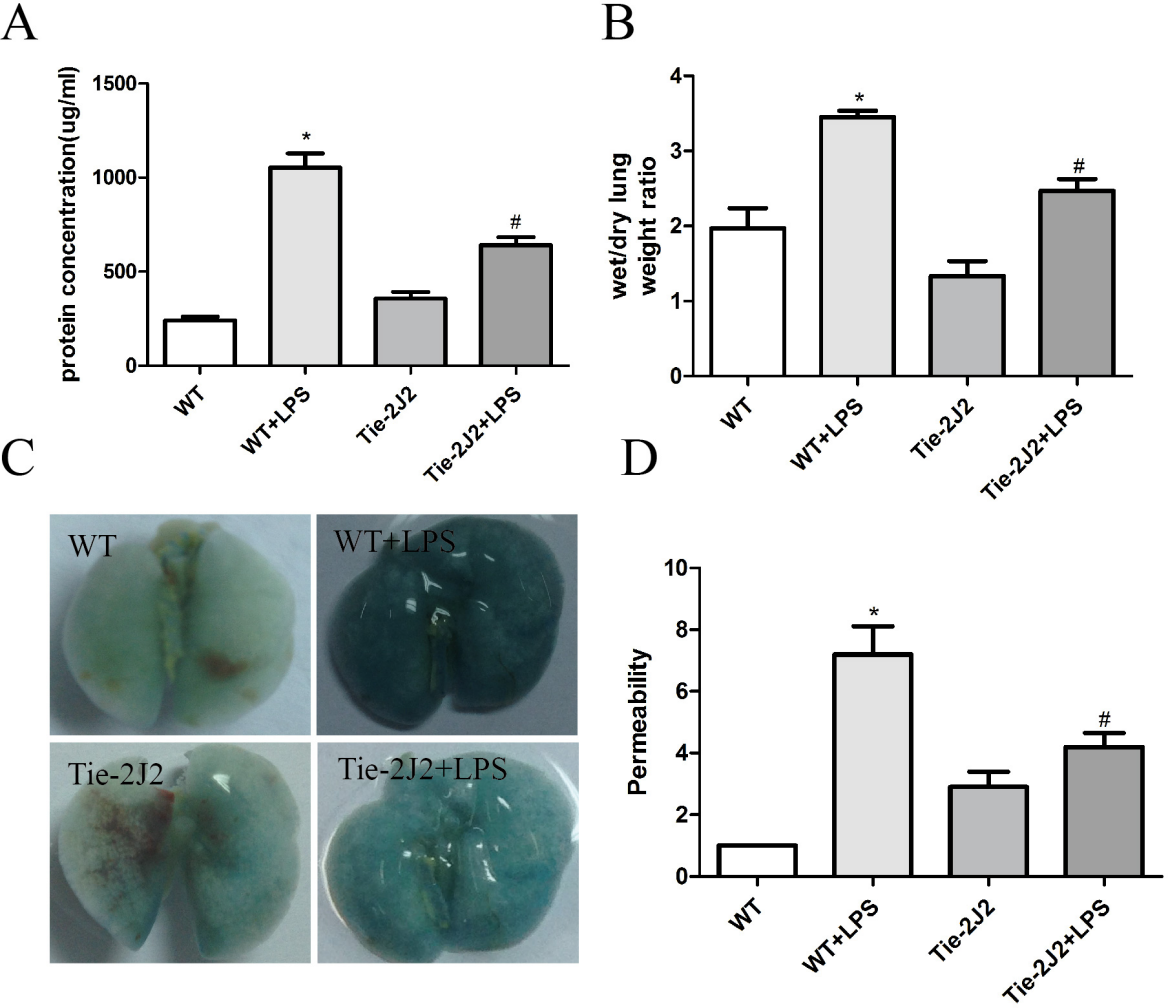
CYP2J2 overexpression reduced LPS-induced mortality by attenuation of hyperpermeability 12 hours after receiving LPS, mice were sacrificed and pulmonary transvascular albumin permeability in BALF and wet-to-dry lung weight ratios were measured (**A** and **B**). lung vascular permeability was assessed by accumulation of Evans Blue dye in the lungs. Lungs were excised after perfusion and imaged (**C**). Spectrophotometric analysis of Evans Blue stained albumin content in the lung tissues was quantified (**D**). Data are expressed as means ± SEM. *n* = 5 per group. *P < 0.05 versus WT; ^#^*P* < 0.05 versus WT+LPS.

When Lung EC barrier was disrupted, the permeation of fluid and macromolecules into the interstitium and alveolar space are increased. Compared with that of control mice, LPS treatment induced an increase in total protein within the bronchoalveolar lavage fluid (BALF), which is greatly reversed by CYP2J2 overexpression (Figure 2A). Furthermore, the wet/dry weight ratio of lung was also reduced in CYP2J2 transgenic mice (Figure 2B). Moreover, infiltration of albumin from the vessel into the lung tissue induced by LPS injection was also attenuated in CYP2J2 as shown in Figure 2C and 2D.

In addition, TPPU treatment maintained barrier stabilization characterized by decreased lung protein concentration, wet/dry ratio and endothelial permeability in mice treated with LPS as shown in Supplementary Figure 2A–2D. Interestingly, decreased TNF-a and IL-1β levels in BALF were also observed in mice treated with TPPU (Supplementary Figure 3A and 3B). These data indicated that EETs reduced LPS-induced mortality via decreased lung hyperpermeability.

### AUDA prevents LPS induces tyrosine phosphorylation of adherens junction components

**Figure 3:**
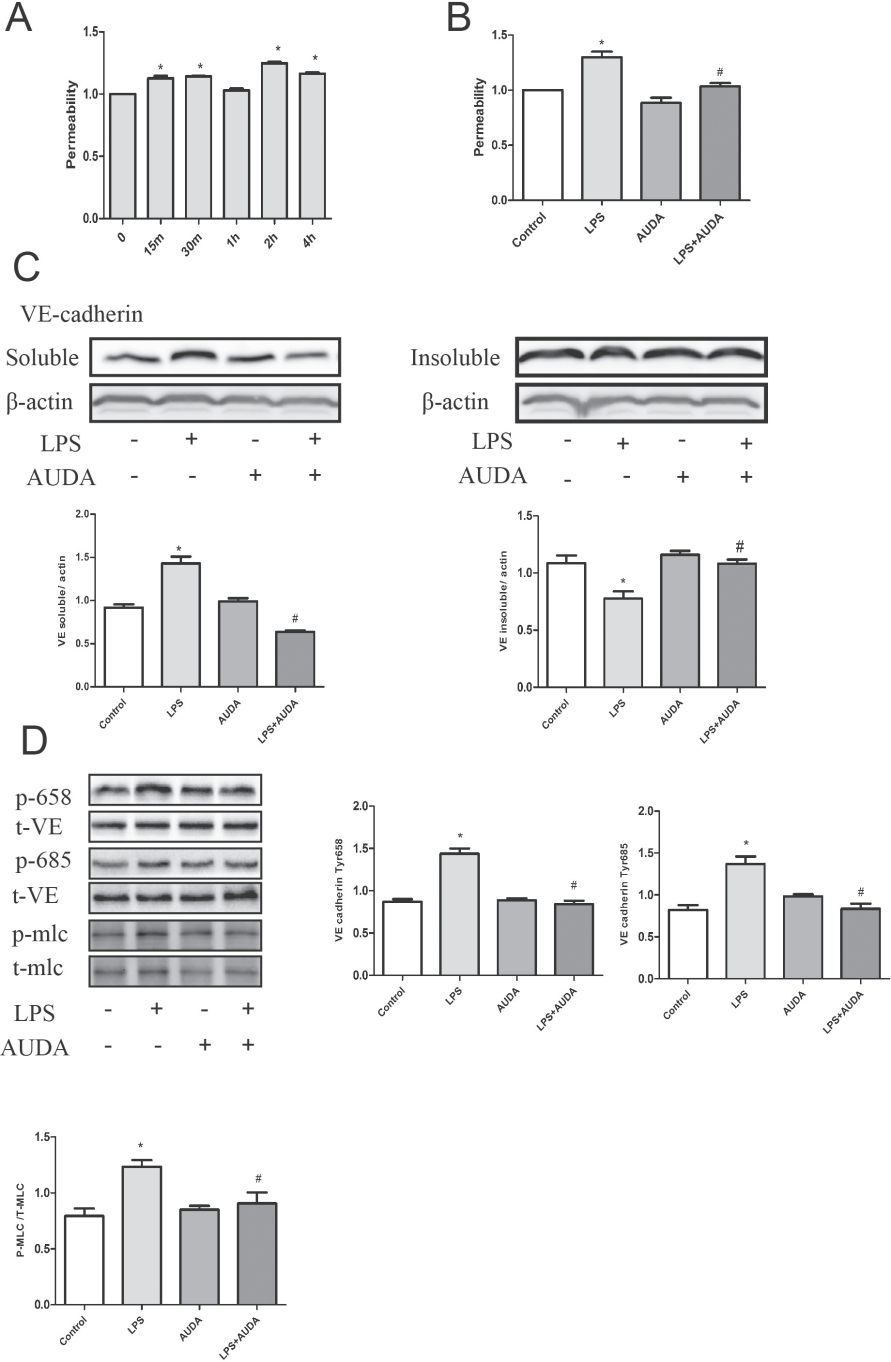
AUDA suppressed LPS-induced hyperpermeability by targeting Adherens Junction Components (**A**) *in vitro* permeability assay that showing the effect of AUDA on vascular barrier function; (**B**) cells were treated with triton and SDS lysis buffer that enables us to obtain the soluble and insoluble contents of cell lysate, expression levels were measured by western blot; (**C**) the effect of AUDA pretreatment on VE cadherin 658 and 685 tyrosine residue as well as MLC phosphorylation. Each tests was repeated for at least three times. Data are expressed as means ± SEM. *P < 0.05 versus LPS; ^#^*P* < 0.05 versus LPS+AUDA.

Compared with transcellular permeability, cultured cells may have lost specialized vesicle shuttling systems, which may be better models for measurements of changes in paracellular permeability [20]. The increase in permeability was obviously observed 2 hours after LPS treatment as shown in Figure 3A, and AUDA treatment prevented the increase in permeability induced by LPS treatment (Figure 3B). When stimulated with LPS, VE-cadherin is phosphorylated and becomes soluble and internalized, which contributes to its disassociation from the opposite. As shown in Figure 3C, LPS induced a shift of VE-cadherin from the insoluble to the soluble fraction which is reversed by AUDA pretreatment. Consistent with this, LPS induced VE-cadherin 658 and 685 tyrosine phosphorylation as well as MLC phosphorylation, and AUDA treatment markedly inhibited the phosphorylation of VE-cadherin 658 and 685 tyrosine and MLC triggered by LPS treatment (Figure 3D).

In addition, 11,12-EET treatment inhibited the increase in permeability induced by LPS challenge (Supplementary Figure 4A), and moreover, 11,12-EET markedly inhibited the phosphorylation of VE-cadherin 658 and 685 tyrosine and MLC triggered by LPS treatment as shown in Supplementary Figure 4B–4G. Apoptosis plays an important role in vascular hyperpermeability [21]. Interestingly, we did not observe any obvious apoptosis in LPS-treated cells (Supplementary Figure 5), indicating that EETs directly suppressed vascular hyperpermeability.

### AUDA inhibited LPS-induced hyperpermeability by targeting Rho-rock pathway

**Figure 4:**
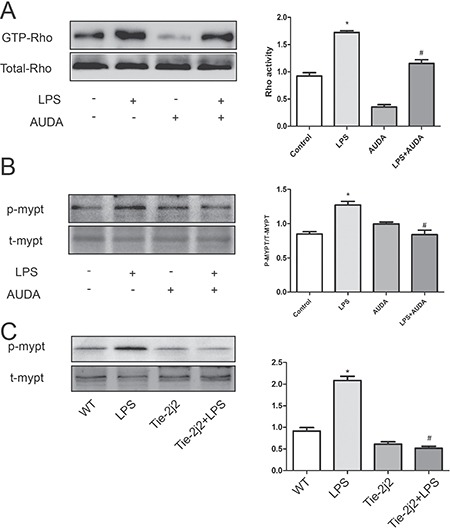
AUDA inhibited LPS-induced hyperpermeability by targeting Rho-rock pathway LPS induced rho activation and subsequent MYPT phosphorylation is attenuated by AUDA pretreatment (**A** and **B**); In LPS-injected mice, MYPT expression was also increased, but CYP2J2 overexpression reversed this trend (**C**). Each tests was repeated for at least three times. Data are expressed as means ± SEM. *P < 0.05 versus control; ^#^*P* < 0.05 versus LPS. In animal tests, *n* = 5. *P < 0.05 versus WT; ^#^*P* < 0.05 versus WT+LPS.

ROCK contains a Rho binding domain, and binding of GTP-bound active RhoA induces conformational changes in ROCK and stimulates ROCK activity [22]. Activation of ROCK in turn phosphorylates various downstream targets to regulate cell motility and gene transcription. One of the best-characterized substrates of ROCK is myosin phosphatase target-1 (MYPT-1) subunit, and phosphorylation of MYPT-1 inhibits myosin phosphatase, an enzyme that dephosphorylates MLC, leading to subsequent myosin L chain (MLC) phosphorylation and stress fiber formation [23, 24]. Previous study revealed that Rho-rock played an essential role in LPS-induced hyperpermeability [25]. LPS treatment markedly increased GTP-Rho and phosphor-MYPT-1 expression, and AUDA administration obviously prevented the increase in GTP-Rho and phosphor-MYPT-1 expression as shown in Figure 4A and 4B. Furthermore, the trendency of GTP-Rho and phosphor-MYPT-1 expression was further confirmed in CYP2J2 transgenic mice as shown in Figure 4C. These data indicated that EETs inhibited LPS-induced hyperpermeability by targeting Rho-rock pathway.

### ROS generation was involved in LPS-induced hyperpermeability

**Figure 5:**
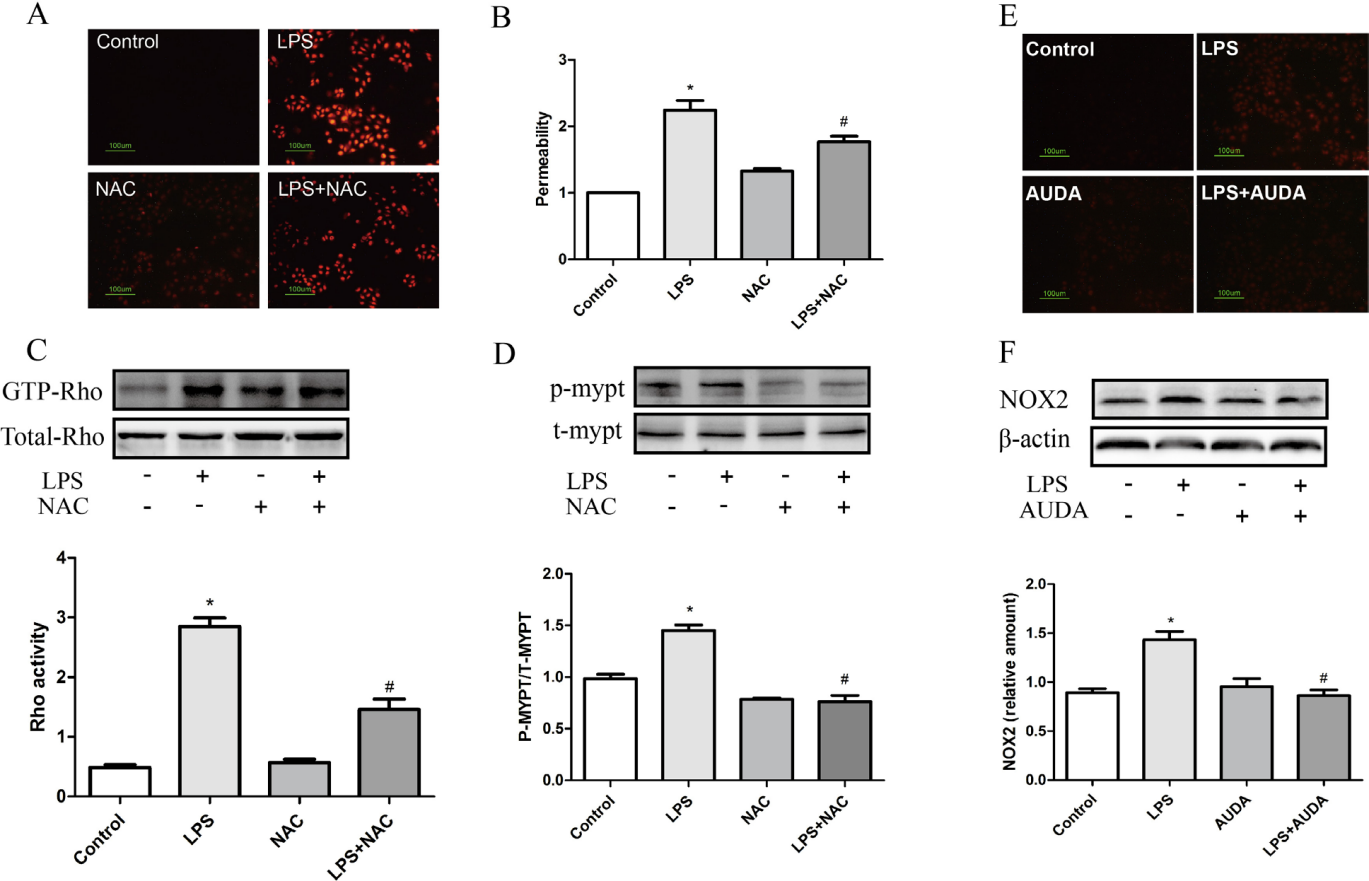
ROS generation was involved in LPS-induced hyperpermeability (**A**) NAC eliminated LPS induced ROS production; (**B**) NAC suppressed LPS induced permeability increase; (**C** and **D**) LPS induced Rho activation and subsequent MYPT phosphorylation was attenuated by NAC. (**E**, **F**) LPS induced ROS production and NOX2 expression was attenuated by NAC. Each tests was repeated for at least three times. Data are expressed as means ± SEM. *P < 0.05 versus Control; ^#^*P* < 0.05 versus LPS.

Endothelial barrier can be impaired by both endogenous and exogenous ROS, via either inducing rearrangements of the endothelial cytoskeleton or stimulating intercellular junctions rupture [26]. Previous study revealed that H2O2 or xanthine/XO both can cause remodeling of the actin filament networks, increasing intracellular tension, inducing intercellular gap formation, and stimulating redistribution of cell junction components such as VE-cadherin [26]. Whether ROS was involved in LPS-induced hyperpermeability was determined. As expected, LPS triggered ROS production, and NAC treatment partly eliminated ROS generation induced by LPS treatment as shown in Figure 5A, and moreover, NAC treatment partly inhibited LPS-induced hyperpermeability (Figure 5B). Interestingly, NAC treatment markedly prevented the increase in GTP-Rho and phosphor-MYPT-1 expression (Figure 5C and 5D). Similarly, AUDA treatment also partly eliminated ROS generation induced by LPS treatment as shown in Figure 5E. In addition, LPS upregulated NOX2 expression in endothelial cells, and AUDA treatment significantly prevented the upregulation in NOX2 expression induced by LPS treatment as shown in Figure 5F. These data indicated that essential role of ROS generation was involved in LPS-induced barrier dysfunction, and EETs partly inhibited NOX2-mediated ROS production.

### AUDA inhibited LPS-induced hyperpermeability by mitigating ROS mediated ROCK activation

**Figure 6:**
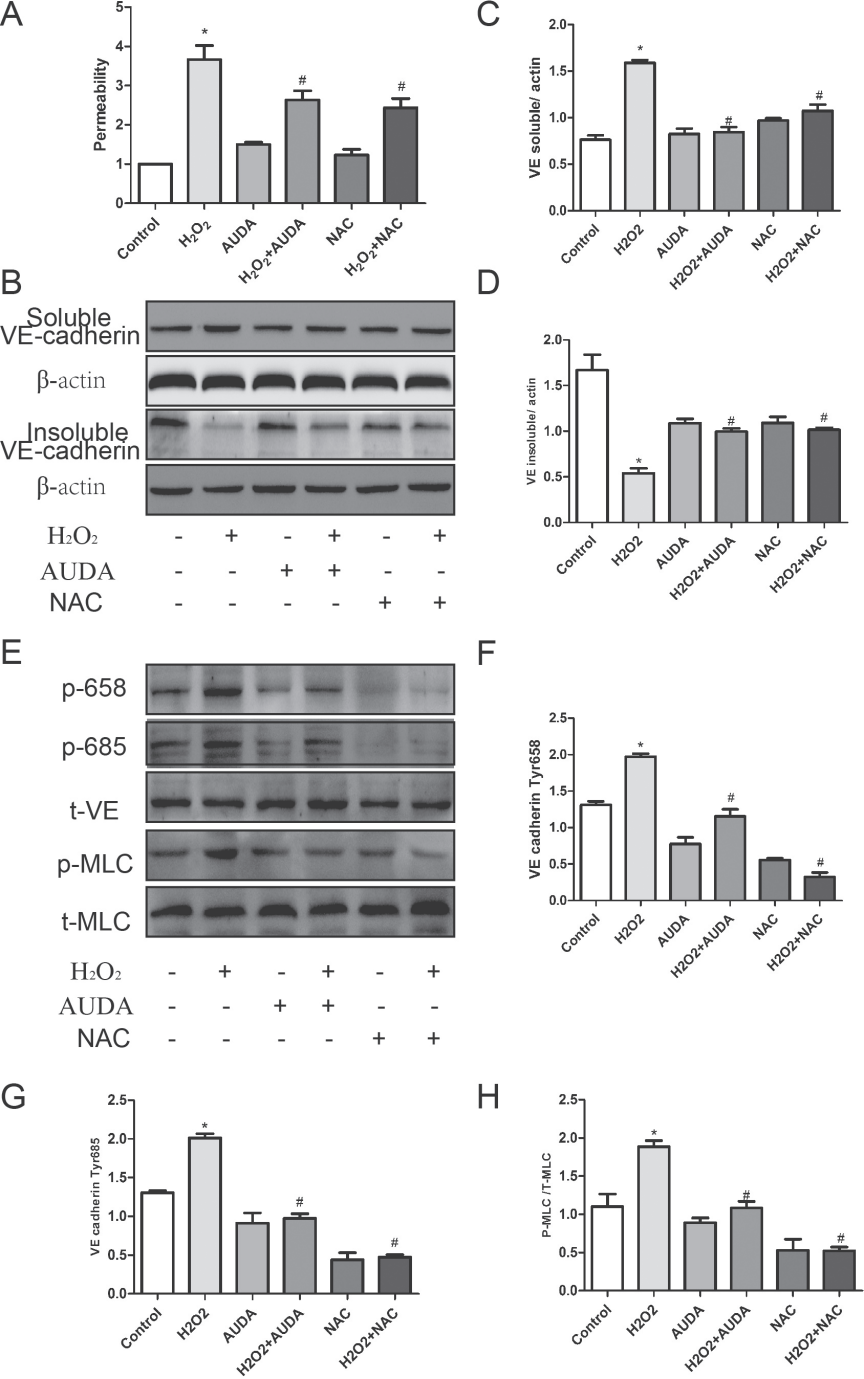
AUDA inhibited LPS-induced hyperpermeability by mitigating ROS mediated ROCK activation (**A** and **B**) AUDA suppressed LPS induced ROS production and NOX2 expression; (**C**) H_2_O_2_ induced permeability increase was attenuated by AUDA; (**D**) AUDA inhibited H_2_O_2_ induced shift of VE-cadherin from insoluble to soluble contents; (**E**) AUDA reduced H_2_O_2_ induced VE- cadherin and MLC phosphorylation. Each tests was repeated for at least three times. (**F**–**H**) the quantity of the protein in Figure E. Data are expressed as means ± SEM. *P < 0.05 versus Control; ^#^*P* < 0.05 versus H2O2.

As described above, EETs partly inhibited NOX2 mediated ROS production. It is speculated that oxidative stress was involved in EETs-regulated endothelial barrier function maintenance. Interestingly, AUDA reduced H_2_O_2_-induced hyperpermeability, which is similar to ROS scavenger NAC treatment as shown in Figure 6A, thus indicating that EETs regulated endothelial barrier function by targeting ROS scavenge. In addition, H_2_O_2_ induced a shift of VE-cadherin from the insoluble to the soluble fraction, which is reversed by AUDA pretreatment (Figure 6B–6D). Consistent with this, H_2_O_2_-induced increase in VE-cadherin 658 and 685 tyrosine phosphorylation as well as MLC phosphorylation was also prevented by AUDA treatment (Figure 6E–6H). These data indicated that EETs inhibited LPS-induced hyperpermeability by mitigating ROS mediated Rock activation.

### AUDA inhibited LPS-induced hyperpermeability by regulating GRP78 mediated SRC activation

**Figure 7:**
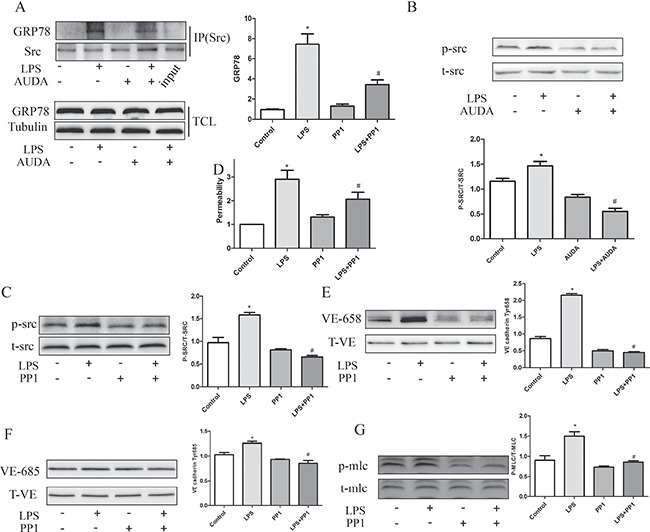
AUDA inhibited LPS-induced hyperpermeability by regulating GRP78 mediated SRC activation (**A**) LPS induced GRP78/Src interaction and Src activation was restrained by AUDA treatment; (**B**) LPS induced permeability increase was reduced by Src inhibitor PP1; (**C**) PP1 decreased LPS induced phosphorylation of VE-cadherin and MLC. (**D**) PP1 attenuated LPS induced permeability increase. (**E**–**G**) LPS induced VE cadherin and MLC phosphorylation was suppressed by PP1 treatment. Each tests was repeated for at least three times. Data are expressed as means ± SEM. *P < 0.05 versus Control; ^#^*P* < 0.05 versus LPS.

Previous study indicated that ISM (Isthmin) reduced VE-cadherin expression on cellular junctions, by mechanism involving a direct interaction between GRP78 and Src, which leaded to Src activation and subsequent adherens junction phosphorylation proteins [27]. Since ISM is upregulated and contributes to LPS-induced acute lung injury and hyperpermeability in mice [27]. GRP78/Src interaction was determined in this study. By using immunoprecipitation, LPS-induced GRP78-SRC interaction and Src activation was attenuated by AUDA incubation (Figure 7A and 7B). In addition, Src inhibitor PP1 decreased LPS-induced hyperpermeability as shown in Figure 7C and 7D. In addition, VE-cadherin and MLC phosphorylation was also attenuated by PP1 treatment as shown in Figure 7E–7G. These data indicated that EETs inhibited LPS-induced hyperpermeability by inactivating GRP78/SRC signaling pathway.

### AUDA inhibited LPS-induced hyperpermeability by regulating GRP78-Src-ROS-RHO-ROCK pathway

**Figure 8:**
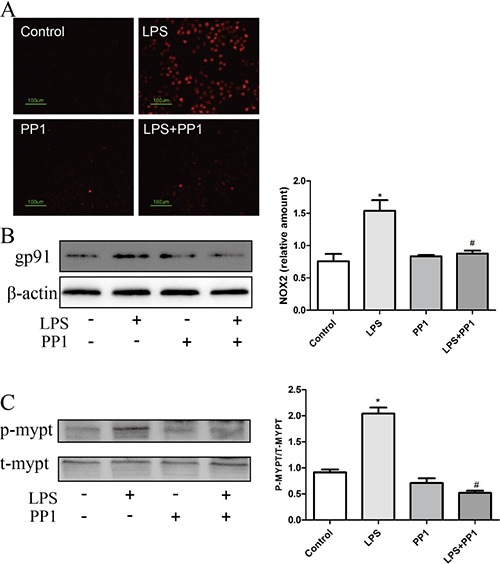
Src acted upstream of ROS-Rho-ROCK pathway (**A** and **B**) PP1 inhibited LPS induced ROS production and NOX2 expression; (**C**) LPS induced MYPT phosphorylation was restrained by PP1 treatment. Each tests was repeated for at least three times. Data are expressed as means ± SEM. *P < 0.05 versus Control; ^#^*P* < 0.05 versus LPS.

Src tyrosine kinases play a critical role in the disruption of cell–cell contacts [28], in order to clarify the concrete mechanism by which Src regulated LPS induced hyperpermeability, we focused on the relationship between Src and ROS. LPS-induced ROS generation and NOX2 expression as well as MYPT phosphorylation was suppressed by Src inhibitor PP1 (Figure 8), which indicated Src as upstream of ROS-Rho-ROCK pathway. Consequently, we may take it granted that EETs inhibited LPS-induced hyperpermeability by regulating GRP78-Src-ROS-RHO-ROCK pathway.

## DISCUSSION

**Figure 9:**
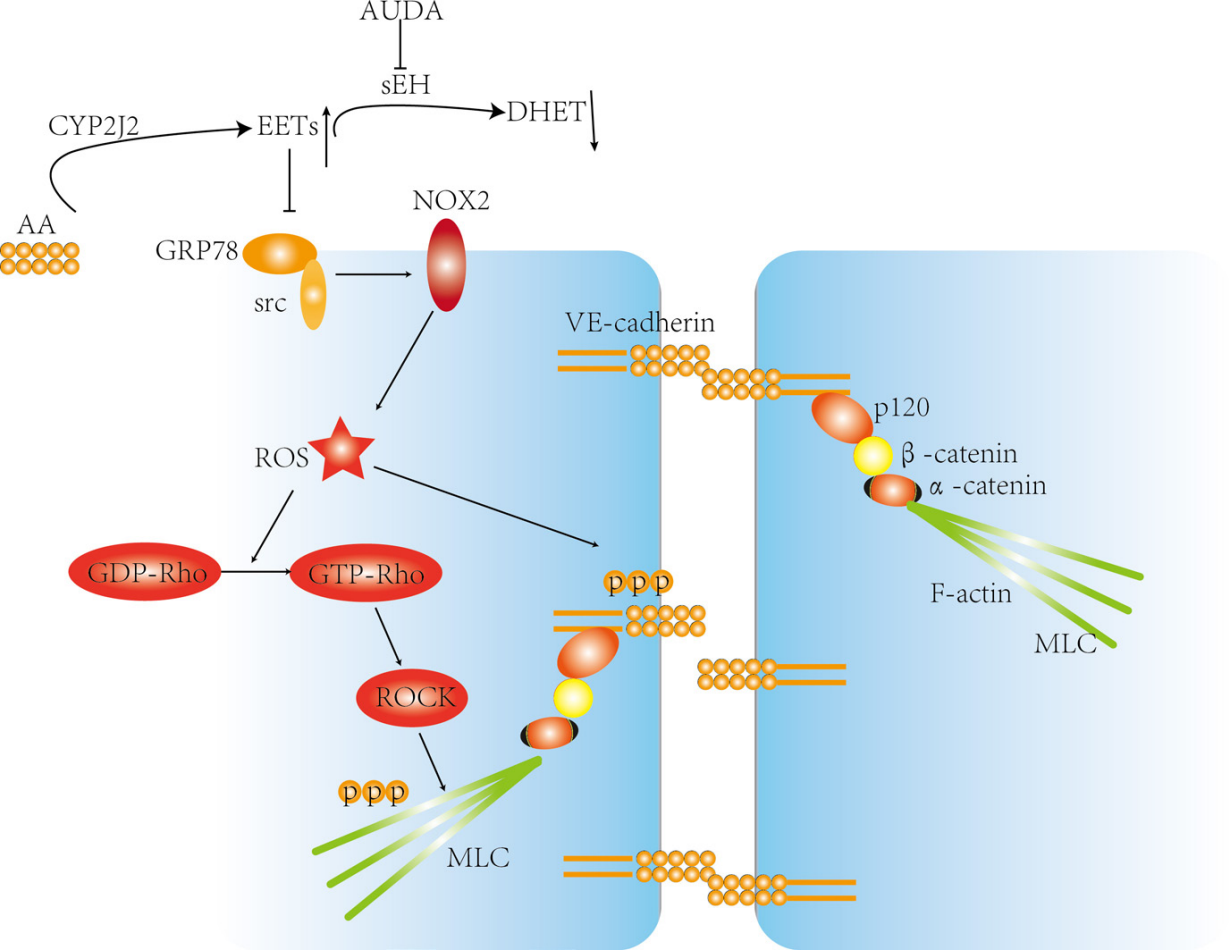
Proposed model about the mechanisms by which CYP2J2 regulated LPS-induced barrier dysfunction AS indicated, LPS promoted GRP78/Src interaction and subsequent activation, leading to increased ROS generation and Rho activation, however, by suppressing GRP78/Src interaction, the CYP2J2-EETs inhibited ROS production and Rho-ROCK activation. At the same time, VE-cadherin and MLC phosphorylation was also interrupted, thus suppressed LPS induced barrier dysfunction eventually.

In the present study, we provided evidence that CYP2J2 overexpression improved survival of septic mice by maintaining endothelial barrier stability and reducing lung hyperpermeability, and its relative mechanisms were associated with decreased VE-cadherin and MLC phosphorylation and increased stability of inter-cellular junctions, which was mediated by attenuation of GRP78/Src/NOX2/ROS/Rho/ROCK signaling pathway (Figure 9). These data indicated that CYP2J2/EETs was a potential target for lung hyperpermeability induced by sepsis.

When suffered infection, the cell junctions of vascular endothelium are destabilized, allowing fluid and cells to infiltrate into peripheral tissues, facilitating clearance of infection and tissue repair [29]. Current therapies attempting at blocking inflammatory cytokine responses proved ineffective at reducing the pathologies associated with sepsis, thus highlighting the need for a new therapeutic strategy targeting at vascular barrier maintenance. In our study, the reduced mortality was accompanied with reduced fluid and molecular infiltration as well as leukocytes into interstitial space, which identified barrier permanence as one of the mechanisms that CYP2J2 improved survival of septic mice, and this is consistent with previous report that increased resistence of ECs to ischemic brain injury in female is due to lower sEH expression [18].

Destabilization of AJs by phosphorylation and internalization of VE-cadherin, together with and increased acto-myosin contractility induced by reorganization of the actin cytoskeleton [6], are two independent mechanisms that break apart the junctions. Previous studies revealed that EETs had heterogeneous impacts on basic filtration coefficiency, which indicated that 5,6-EET and 14,15-EET increased lung capillary K (f,c), whereas the 8,9-EET and 11,12-EET treatment did not show any difference compared to baseline [30]. However, whether EETs affect lung permeability revoked by LPS treatment remains largely unknown. In this study, both AUDA and 11,12-EET treatment reduced LPS-induced VE-cadherin and myosin light chain (MLC) phosphorylation, and subsequently attenuated lung hyperpermeability. In addition, the endothelial apoptotic cascade is an important underlying mechanism of capillary leakage. In this study, however, we found that LPS did not induce apoptosis of HUVECs in the experimental condition. Interestingly, pericytes, marked by NG2 expression, which also control integrity of vascular integrity, were increased in CYP2J2 transgenic mice, indicating that re-attracting pericytes to micro-vessels may be one of the possible mechanisms by which CYP2J2 overexpression decreased lung hyperpermeability, but it still needs further investigation.

Among the members of the Rho family, RhoA, Rac and Cdc42 have been characterized for their important role in regulation on endothelial permeability through targeting distinct subcellular signaling molecule [31]; RhoA plays in the assembly of adherens junctions and stress fiber formation that stimulates endothelial cell contractility by controlling the phosphorylation state of myosin light chain (MLC) [22], whereas Rac and Cdc42 have been shown to promote formation of lamellipodia and filopodia [6]. Targeting their common terminal effectors (RhoA/ROCK) may represent a promising strategy against vascular injury. However, basal Rho activity is also essential for maintaining endothelial barrier function. The Rho GTPase activities at sites of cell-cell contacts is regulated by multiple mechanisms. LPS has also been shown to induce Rho/Rho kinase-dependent MLC phosphorylation and stress fiber formation, which increases actin filament cross-linking and produces a contractile force that results in increased vascular permeability in cardiovascular sepsis shock [22]. In our study, we observed that AUDA reduced LPS-induced Rho activation and Rock activity, which is in line with previous study which demonstrated that EETs suppressed ROCK activation in pulmonary ECs(reference). Based on the results described above, it is speculated that EETs preserved endothelial barrier homeostasis by targeting Rho-rock pathway.

Previous data indicated that Src have been shown to contribute to endothelial cell hyperpermeability [28, 32]. Inhibition of Src by PP2 or knockdown of SFK members partially protected against LPS-mediated barrier disruption [28]. In addition, expression of constitutively active pp60 Src has been shown to decrease endothelial barrier function [33]. Therefore, we tested whether inhibition of Src would decrease the LPS- mediated RhoA activation. Our data clearly show that pretreatment with the Src inhibitor, PP1, completely blocked the LPS-induced RhoA activation characterized by MYPT phosphorylation, and decreased phosphorylation of MLC and VE-cadherin induced by LPS treatment in HUVECs. Taken together, these data indicated that LPS-induced RhoA activation is mediated by Src activation.

There is substantive evidence that oxidative stress increases the permeability of the endothelial monolayer to fluids and macromolecules. Previous study revealed that ROS produced by activated leukocytes and endothelial cells have been implicated in endothelial contraction and barrier disruption [34, 35]. In addition, NOX4 derived ROS can activate Rho-rock signaling pathway [36]. HUVECs only express NOX2 on the cell membrane [26] and 80% of the ROS production and in the isolated lung is originated from NOX2 activation [37]. Previous study revealed that MJ33 inhibited LPS-induced hyperpermeability via reduced ROS production, confirming the role of ROS generation in barrier disruption [37]. In our study, AUDA treatment prevented LPS-induced Rho activation, which was attributed to reduced ROS generation.

Since LPS-induced Rho activation is mediated by ROS generation and concomitantly by Src activation, some other study demonstrated that Src activation promoted mitochondrial ROS generation [38, 39] and selective silencing of c-Src diminished LPS-induced tyrosine phosphorylation and barrier disruption [40]. In our study, PP1 treatment prevented LPS-induced ROS generation and Rho activation, which identified that Src acted upstream of ROS-induced activation of Rho-rock signaling pathway.

The 78 kDa glucose-regulated protein(GRP78) always reside in endoplasmic reticulum, which is generally regarded as a stress induced chaperone regulating the strict maturation process of nascent glycoproteins [41]. However, recent studies reported that GRP78 also existed on cell surface, where it usually binds to a variety of ligands and functions as signal-transducing receptor [42]. Previous studies revealed that a direct interaction of GRP78 with Src by ISM leaded to Src activation and subsequent phosphorylation of the constituent of cell junctions such as adherens. Blocking cell-surface GRP78 significantly alleviates LPS-induced pulmonary vascular hyperpermeability in mice [27]. In this study, LPS treatment increased the interaction of GRP78 with Src and subsequent Src activation, and subsequently promoted VE-cadherin and MLC phosphorylation, which was markedly attenuated by sEH inhibitor AUDA addition.

The results presented in the study demonstrated that EETs reduced Src activation via reduced GRP78-Src interaction, and subsequently inhibited ROS generation, Rho-ROCK activation, contributing to reduced VE-cadherin and MLC phosphorylation, and eventually increased vascular stability, which was accompanied by improved survival of septic mice. These data indicated that CYP2J2/EETs was a potential target for lung hyperpermeability induced by LPS treatment, which may contribute to the development of new therapeutic approaches for pulmonary edema and other diseases caused by abnormal vascular permeability.

## MATERIALS AND METHODS

### Animals

Endothelial specific CYP2J2 (Tie2J2) transgenic mice (from Dr. Darryl C. Zeldin, NIEHS, Research Triangle Park, North Carolina, USA) and their wild-type (WT) genetic controls were used. The mice were housed in temperature-controlled cages under a 12-hour light-dark cycle and were given free access to water and normal chow. All procedures were approved by the Animal Research Ethics Board at Huazhong University of Science and Technology and conformed to Directive 2010/63/EU of the European Parliament. Mice aged 8 to 12 weeks were used for subsequent experiments. A solution of LPS (Sigma, 15 mg/kg) in 0.9% saline was delivered intraperitoneally; 0.9% saline alone was used as a control. After 6 hours, bronchoalveolar lavage was performed by intratracheal injection of 0.5 mL of PBS solution for three times, followed by gentle aspiration. The recovered fluids were processed for determination of total protein concentration and cytokines assay. When we used TPPU (Toris) to inhibit the hydrolysis of EETs, TPPU was Intragastric administrated for a week ahead of the LPS injection. Lungs from challenged mice were collected for histological evaluation or were frozen at minus 80°C.

### Evans blue staining in lungs

Accumulation of Evans Blue dye in lung tissue was evaluated according to a protocol described previously [43]. In brief, having been injected LPS (15 mg/kg) introperitoneally for 6 hours, the mice were injected with Evans Blue dye (Sigma, 20 mg/kg) into the external jugular vein. 30 minutes later, a thoracotomy was performed and the lungs were perfused with 0.9% saline containing heparin, to remove blood. Both left and right lobes of the lung were excised. Evans Blue accumulation in the lung tissue was measured by spectrofluorometric analysis of lung tissue lysates.

### Enzyme-linked immunosorbent assay

The TNF-α and IL-1β in the bronchoalveolar lavage was measured by ELISA assay (Neobioscience) according to the instructions.

### Immunofluorescence microscopy

Having been washed using PBS for three times, cells were fixed with 4% formaldehyde freshly prepared from paraformaldehyde (PAF) and permeabilized with 0.5% Triton X-100 for labeling with NG2 and CD31 antibodies(CST, cell signaling technology). After incubation with the first antibody overnight, the secondary fluorescence labeled antibody(Boster) was followed. Eventually, cells were treated with DAPI and then pictures were taken.

### Cell layer permeability assay

Transwell units with 6.5-mm diameter and 0.4-μm pore size polycarbonate filters that are coated with human fibronectin (Toris) were used. Culture medium in the upper and lower compartments was 100 and 600 uL, respectively, as suggested by the manufacturer. After confluence, cells were incubated with 11,12-EET or AUDA, immediately followed by LPS (1 mg/ml) before addition of fluorescein (FITC)-conjugated dextran (38 900 Da, final concentration 1 mg/mL, (Sigma)) to measure permeability. At the indicated time points, a 50 μL sample was removed from the lower compartment, fluorescence content was measured in a fluorimeter at 492 nm absorbance and 520 nm emission wave length, respectively [44].

### Determination of ROS formation

ECs were washed with PBS and incubated in 20 μmol/L of dihydrorhodamine (Sigma) with 0.5% FBS for 30 min. At the end of incubation, the buffer was aspirated and the cells were washed with 1 × PBS and then were imaged.

### Rho activity asssay

Rho activation was evaluated using a Rho activation assay kit (Neweast Bioscience) according to the manufacturer's instructions.

### Western blot analysis

Western blotting was performed as described previously [45], CYP2J2 antibody was prepared by our laboratory. Antibodies including MPO, Total-VE cadherin, NOX2 (gp91), Total-Src and phosphorylated Src are purchased from CST (cell signaling technology). Antibodies including VE-cadherin Tyr-658, VE-cadherin Tyr-685 site, T-MLC, phosphorylated-MLC, T-mypt, phosphorylated-mypt and GRP78 antibody were from Abcam.

### Determination of lung wet-to-dry weight ratio

At the end of the animal experiment, thoracotomy was performed and lungs were exposed. Lungs were excised and immediately weighed to get the wet weight. And then the lungs were dried in an oven at 60°C for 3 days to get the dry weight. Lung dry weight to wet weight ratio was calculated.

### Immunoprecipitation (IP)

IP was performed as described previously [46], In IP experiments, following binding with the primary antibody, residual lysate was collected and probed for alpha-tubulin (CST) for protein loading control. Band intensities were quantified using Image J software

### Statistical analysis

All data were shown as mean ± SEM (Standard Error of Mean). Comparisons between groups were performed by a one-way analysis of ANOVA with *post hoc* analyses performed using the Student-Newman-Keuls method and the level for significant statistical differences was set at *P <* 0.05. Survival rates were assessed by log-rank test.

## SUPPLEMENTARY MATERIALS FIGURES AND TABLES


