# Supplementary material for: EETs reduces LPS-induced hyperpermeability by targeting GRP78 mediated Src activation and subsequent Rho/ROCK signaling pathway
Source: Oncotarget. 2017 Apr 21;8(31):50958–71. doi: 10.18632/oncotarget.17331 (PMC5584221; doi:10.18632/oncotarget.17331)
Supplement: Supplementary file 1 [file oncotarget-08-50958-s001.pdf]

EETs reduces LPS-induced hyperpermeability by targeting GRP78 mediated Src activation and subsequent Rho/ROCK signaling pathway

Supplementary Materials

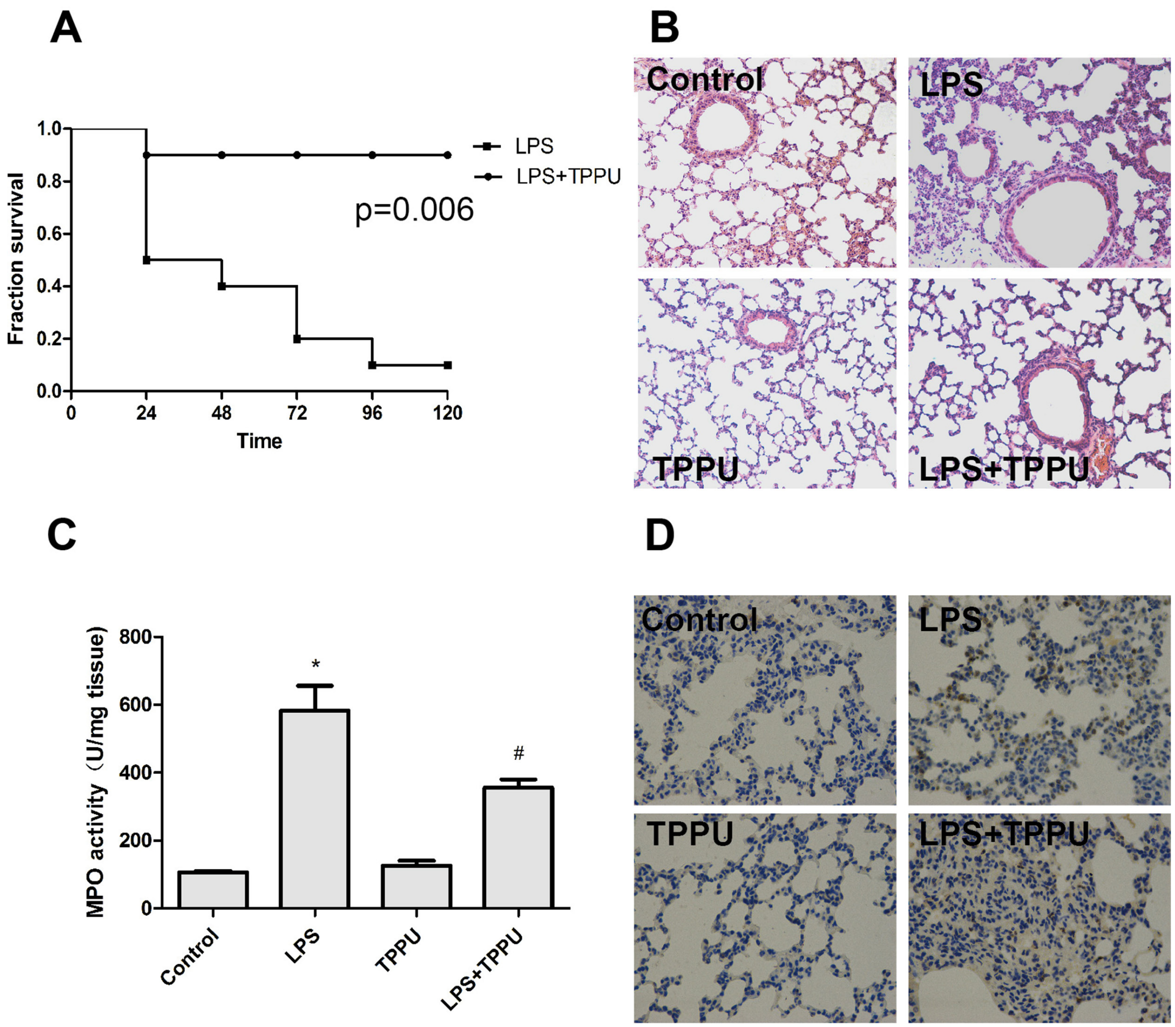

Supplementary Figure 1: sEH inhibitor TPPU reduced sepsis induced mortality. (**A**) survival curve of mice in 96 hours after LPS treatment; (**B**) HE staining of lungs that indicates leukocytes infiltration; (**C**) Determination of MPO activity in lung tissues; (**D**) immunohistochemistry staining of lung tissue MPO expression that indicates neutrophils infiltration. In survival curve test, we use 10 mice in each group, while for other test, $n = 5$. *$P < 0.05$ versus WT; $^{\#}P < 0.05$ versus WT+LPS.

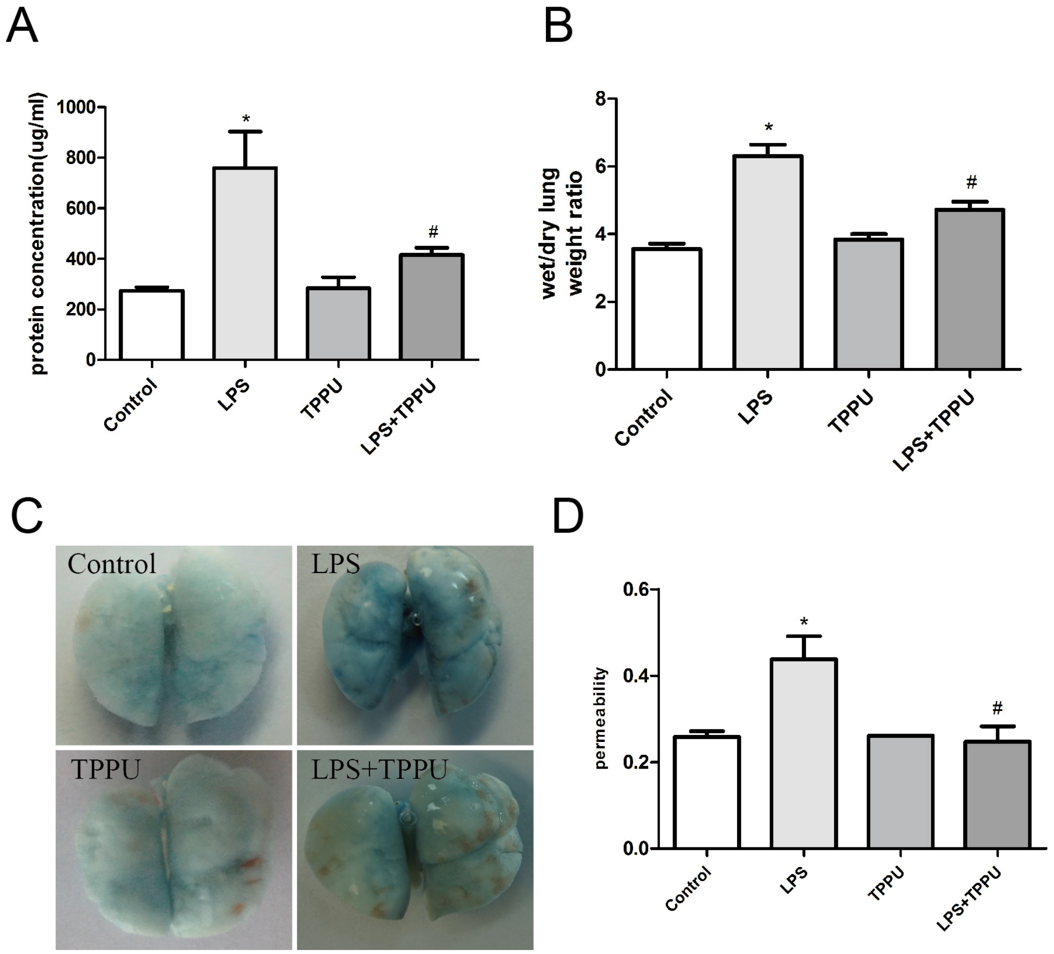

Supplementary Figure 2: sEH inhibitor TPPU reduced LPS induced mortality by attenuation of permeability increase. (**A** and **B**) pulmonary transvascular albumin permeability in BALF and wet-to-dry lung weight ratios were measured. (**C**) lung vascular permeability was assessed by accumulation of Evans Blue dye in the lungs. Lungs were excised after perfusion and imaged. (**D**) Spectrophotometric analysis of Evans Blue stained albumin content in the lung tissues was quantified. Data are expressed as means ± SEM. $n = 5$ per group. *$P < 0.05$ versus WT; $^{\#}P < 0.05$ versus WT + LPS.

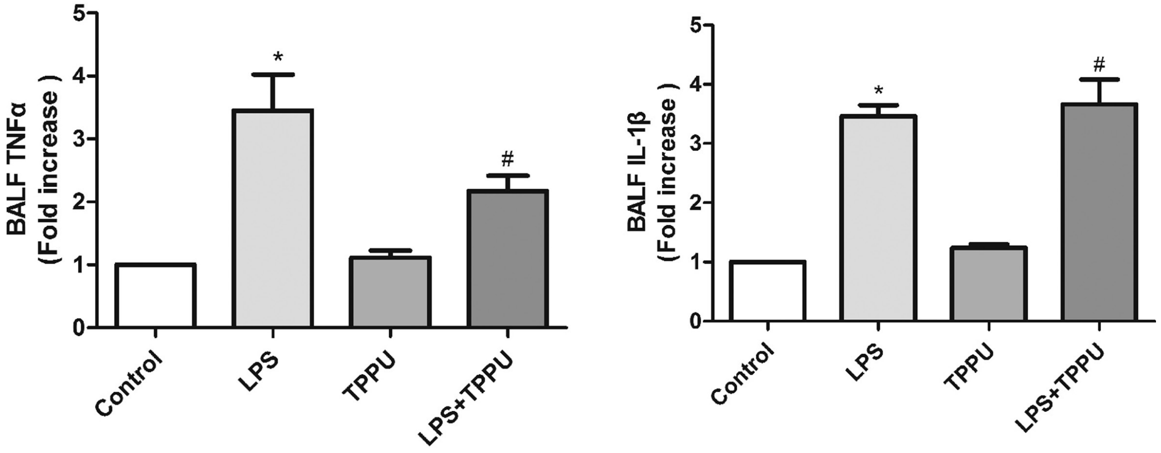

Supplementary Figure 3: ELISA of TNF-α and IL-1β in BALF. (**A**) The level of TNF-α in BALF; (**B**) The level of IL-1β in BALF; Data are expressed as means ± SEM. $n = 5$ per group. *$P < 0.05$ versus WT; $^{\#}P < 0.05$ versus WT + LPS.

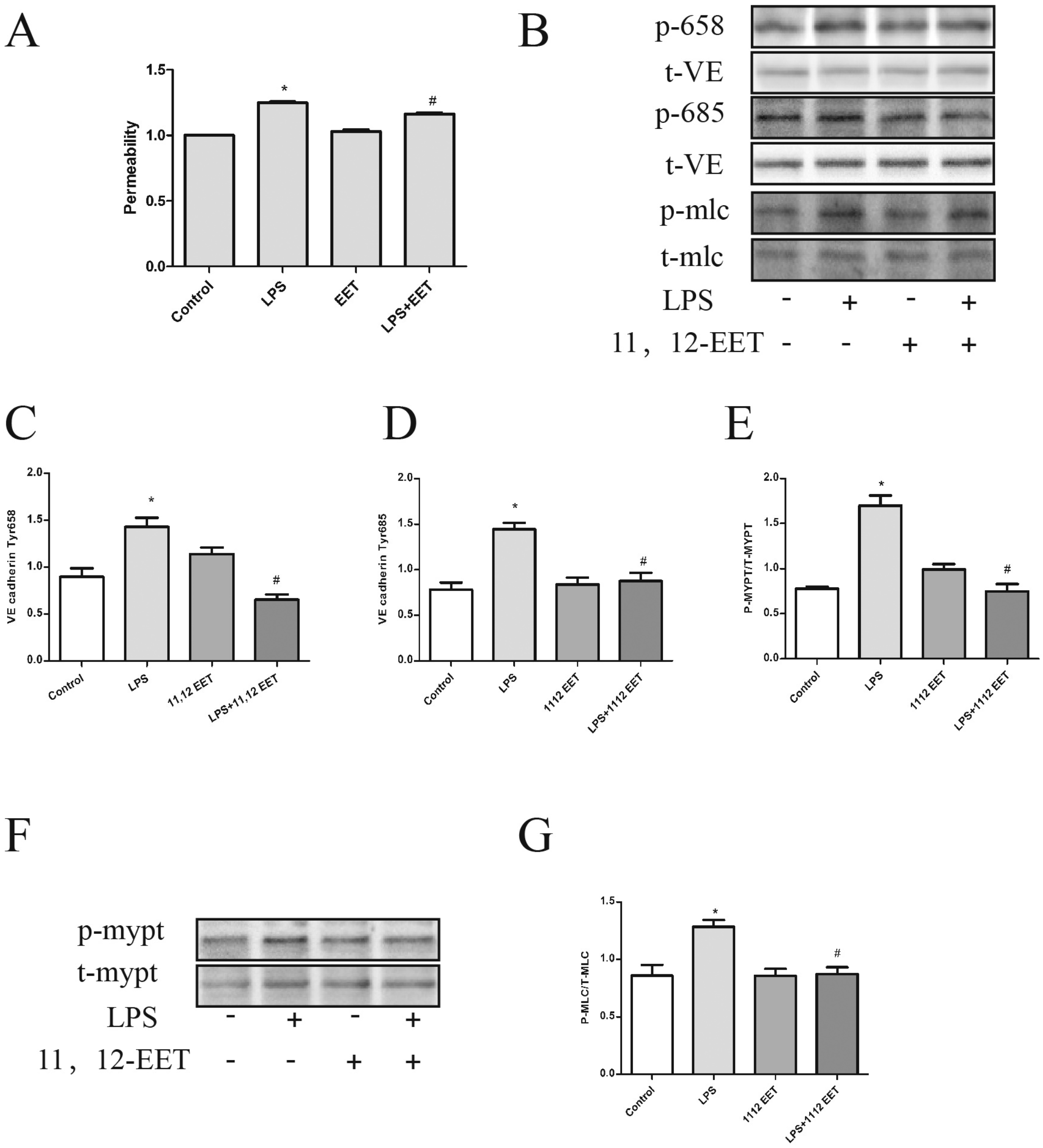

Supplementary Figure 4: 11,12-EET suppressed LPS-induced hyperpermeability by targeting Adherens Junction Components. (**A**) *in vitro* permeability assay that showing the effect of 11,12-EET on vascular barrier function; (**B**) the effect of 11,12-EET pretreatment on VE cadherin 658 and 685 tyrosine as well as MLC phosphorylation. (**C**) LPS-induced MYPT phosphorylation was attenuated by 11,12-EET treatment. Each tests was repeated for at least three times. Data are expressed as means ± SEM. *$P < 0.05$ versus LPS; $^{\#}P < 0.05$ versus LPS + AUDA.

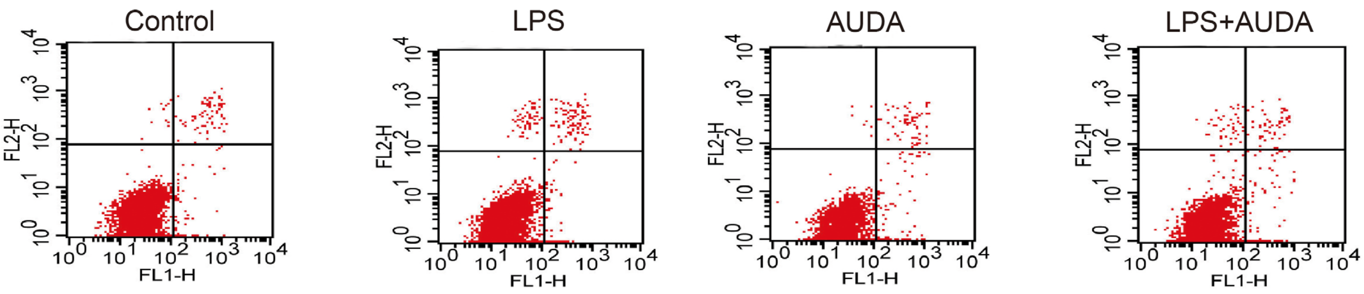

Quad	Control % Gated	LPS % Gated	AUDA % Gated	LPS+AUDA % Gated
UL	0.26	1.09	0.28	1.03
UR	1.89	2.12	1.59	1.37
LL	95.64	95.17	95.27	95.23
LR	2.21	1.62	2.86	2.37

Supplementary Figure 5: Role of endothelial apoptosis in LPS-induced hyperpermeability. LPS treatment did not induce endothelial apoptosis, and moreover, AUDA had no obvious effects on endothelial cells treated with LPS.
